# Correlation of dental pain and pain catastrophizing with oral health status among physically disabled

**DOI:** 10.4317/jced.60816

**Published:** 2023-10-01

**Authors:** Jummala Sasikala, Jagadeeswara-Rao Sukhabogi, Dolar Doshi, Turaga-Sai Susmitha, Aishwarya-Lakshmi Billa

**Affiliations:** 1 Department of Public Health Dentistry, Government Dental College & Hospital, Hyderabad, Afzalgunj Road, near police station, Hyderabad, Telangana, India

## Abstract

**Background:**

Physical disability results in impaired mobility, leading to increased dependency on others and may also have a negative impact on ones general and oral health. Moreover, such individuals could be at a greater risk of being prone to chronic pain conditions. A person’s ability to cope with pain is a consistent and one of the most important predictors of clinical outcome. Catastrophization is known to be a maladaptive coping behaviour that could negatively influence such outcomes.

**Material and Methods:**

A cross-sectional study was conducted among 229 physically disabled individuals at Home for Disabled, Bansilalpet, Secunderabad. Extent of Physical disability was measured using Barthel index of Activities of Daily Living (ADL), type and severity of dental pain was assessed using the short form McGill Pain Questionnaire and catastrophizing using the Pain Catastrophizing Scale (PCS). Dentition status and periodontal status were assessed using the World Health Organization assessment form.

**Results:**

Caries prevalence of the population was 39.7% with a mean DMFT score of 2.8±4.09. Pain catastrophizing showed positive significant (*p*<0.05) correlation with sensory pain(r=0.182), visual analog scale(r=0.168), pain severity(r=0.161) and DMFT (r=0.4). It had significant negative correlation with ADL and bleeding gums.

**Conclusions:**

In this study it was apparent that irrespective of the dependency levels, dentition status had a significant effect on pain catastrophizing level of the individuals.

** Key words:**Disability, Oral Health, Catastrophizing.

## Introduction

Disabled people form a fundamental unit of the community, and it is estimated that approximately 500 million people worldwide are with disabilities (1). According to the National Sample Survey Organization Report, there are an estimated 26.8 million people with disabilities living in India, accounting for 2.21% of the country’s overall population—an increase from 2.13% was observed over the last decade (2).

According to World Health Organization, disability is defined as an umbrella term for “impairments, activity limitations, and participation restrictions denoting the negative aspects of the interaction between an individual (with a health condition) and that individual’s contextual (environmental and personal) factors.” Disability is neither simply a biological nor a social phenomenon (3).

“The Persons With Disabilities Act, 1995” asserts that it is the responsibility of the state towards protection of rights of persons with disabilities; provision of medical care, education, training, employment, and rehabilitation. People with disabilities deserve the same opportunities for oral health and hygiene as those who are healthy, but unfortunately dental care is the most common unmet health care need of the disabled people (4). Physical disability results in impaired mobility, increase the dependency of the disabled on others and may also have a negative impact on general and oral health.

Oral health is a vital part of general health and has significant influence on quality of life of adults, older persons, children, adolescents and their families. Oral health is defined as the ability to speak, smile, smell, taste, touch, chew, swallow and convey a range of emotions through facial expressions with confidence and without pain, discomfort and disease of the craniofacial complex (5). Chewing, swallowing, speechadn acute or chronic pain are some of the quality of life dimensions influenced by oral conditions such as dental caries, periodontitits etc. Several studies have shown that people with disabilities especially those with associated motor disability, present worse oral hygiene and periodontal condition than people without a disability (6-8).

Factors that contribute to poor oral hygiene among people with disability include inadequate brushing technique usually manifested through decreased manual dexterity and absence of care or lack of adequate training to the caregiver (9). Anders and Davis suggested that training dentists, more preventive strategies, and interventions to increase routine oral care as strategies among this group (10).

Moreover, individuals with physical disability may also be at a greater risk for chronic pain conditions and often experience pain soon after onset of impairments. All these factors might lead to poor oral health as reported by Orsos M *et al*. (11) and may lead to an increase in pain experience among individuals.

Usually, how individuals cope with pain is relatively a consistent predictor of important clinical outcomes that includes pain severity and pain related disability, which might be negative or maladaptive coping strategies (12). One of such coping strategies is Catastrophizing which is referred to “an exaggerated negative mental set brought to bear during actual or anticipated painful experience” specifically includes experience of ruminating on pain related thoughts, exaggerating pain experiences and feeling inability to cope with pain (12).

Pain catastrophizing not only can directly influence individual’s experience of pain but it also plays an indirect role in their avoidance behavior. Studies (13,14) have shown that when receiving a dental hygiene treatment, patients with a greater tendency toward pain catastrophizing reported greater pain and fear related experience (including dental care related anxiety and fear).

However, majority of the studies (15-18) have been focused only on oral health status among the disabled individuals. To the best of our knowledge there is no data present till date related regarding dental pain, pain catastrophizing among physical disabled Individuals in India.

Apart from leading to heightened pain perception i.e, making actual pain experience more intense and debilitating, catastrophizing also limits functioning, impacts mental health, influences treatment adherence, increases social isolation and interferes with rehabilitation or therapy. All these are more profound in disabled individuals.

Hence against this background the present study aims to assess dental pain, pain catastrophizing and oral health status among physically disabled.

## Material and Methods

A cross-sectional study was conducted among the physically disabled individuals at Home for Disabled, Bansilalpet, Secunderabad. Ethical approval for the study was obtained from Institutional Ethics Committee of Osmania Medical College (Ref.No.IEC/OMC/2022/M.No.(8)/Acad-82). All the study participants were assured of confidentiality and anonymity. With an expected proportion of 18.18% (based on prevalence of dental caries) and with a precision of 5% at a confidence interval of 95% the minimum sample size of 229 was required. Upon agreement and explanation of the study, written informed consent was obtained from all the study subjects before clinical oral examination.

Subjects who were physically disabled residing at Home for Disabled, Bansilalpet, Secunderabad were included. Subjects who declined to participate in the study, who were not able to answer the questions, who were intellectually disabled (with sensory impairment) and with major compromising medical condition such as cardiac problems were excluded.The survey tool was a questionnaire divided into four sections. The first section of the recorded the demographic details (participants name, age and gender). The second section comprised of Physical disability which was measured using Barthel index of Activities of Daily Living (ADL) (25) (10 item). Type and severity of pain was assessed using the short form Mc Gill Pain Questionnaire (26). The third part incuded Pain Catastrophizing Scale (PCS) (13 item) (27). The fourth section of the questionnaire was to register clinical oral examination (28).

## Results

A total of 229 people took part in the survey, with 148 (64.6%) being female and 81 (35.4%) being male. Half of the sample population (50.2%) was between the ages of 15 and 36 (Table 1). The majority of individuals (83% on the Barthel Activities of Daily Living Index) were moderately dependent. In addition, 80.8% of participants reported walking for more than 50 yards with the assistance of one person (verbal or physical) (Item 9). The study population’s mean ADL score was 80.5±12.5.

**Table 1:**
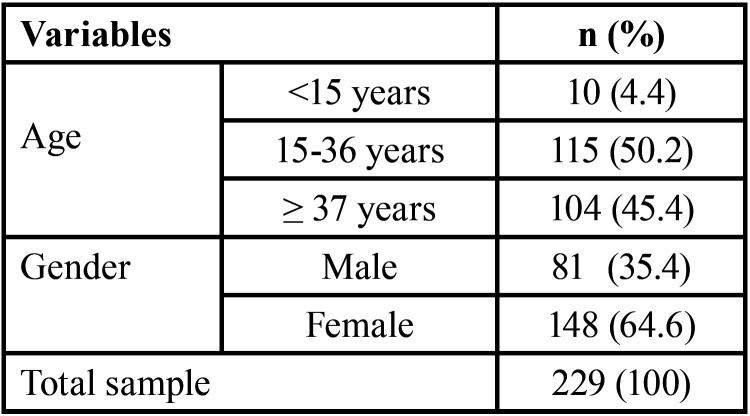
Demographic Details of the Study Population.

Around 68.6% of the individuals reported no pain, and the remaining participants (31.4%) reported only sensory pain. As none of the individuals experienced affective pain, the overall pain score was accounted for solely by sensory pain, which was 1.33±3.15. Sensory pain mean scores were significantly higher in the older age group (37 years). ((≥ 1.7±2.9). Surprisingly, 13.1% of the 38.4% of participants reported excruciating type of pain. The sample’s total mean pain severity score was 1.1±1.8.

There was a statistically significant variation in pain severity based on age (*p*=0.000). The study population’s total mean VAS score was 1.23±2.05 The difference in VAS scores depending on age was statistically significant (*p*=0.001). About 95.2% of the study population had no catastrophizing, while 4.8% of the study population had a significant level of catastrophizing. The overall PCS mean of the sample was 5.8 ± 9.8. Based on age a statistically significant difference was observed among all components of PCS i.e., Ruminification (*p*=0.001), Magnification (*p*=0.002) and Helplessness (*p*=0.001).Compared to the slight and severe dependency groups, higher mean scores of DMFT (3.0±4.2) and its component scores (D= 2.2±3.0, M=0.7±2.8 & f= 0.0±0.07), bleeding (30.97±3.6) and periodontal pockets (4-5mm, >6mm) (0.2±1.4, 0.0±0.4) were observed among the individuals belonging to moderate ADL dependency level which were not statistically significant.

Individuals with higher PCS demonstrated significantly higher means (3.7±3.3) (*p*=0.041) decayed teeth scores in comparison to individuals without pain catastrophizing. A significant positive correlation was observed between higher DMFT values and higher PCS levels (r=0.45, *p*=0.000), whereas bleeding of gums was negatively correlated with sensory pain (r=-0.13, *p*=0.049) and PCS (r= -0.194, *p*=0.004). Periodontal pocket of less than 6mm was negatively correlated with ADL scores (r= -0.135, *p*=0.046).Age (*p*=0.001) of the study participants, their VAS scores (*p*=0.005), pain severity (*p*=0.02), types of pain (*p*=0.006) and PCS levels (*p*=0.000) all were significant predictors of Dental Caries (Table 2).

**Table 2:**
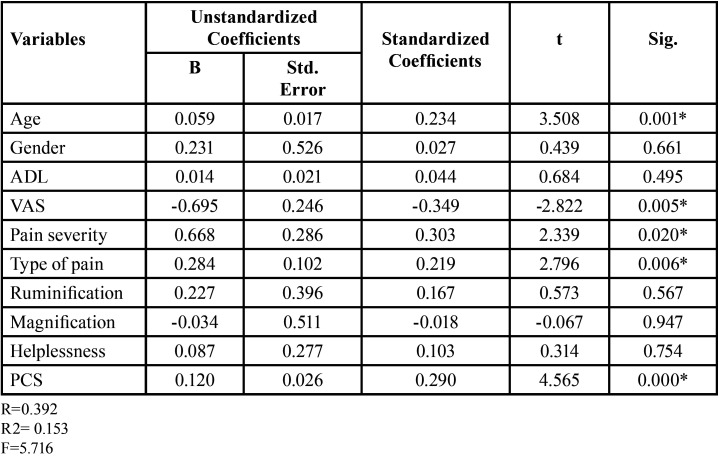
Linear Regression analysis for Dental Caries (Overall DMFT).

Whereas age (*p*=0.008), VAS scores (*p*=0.03) and Helplessness (*p*=0.004) a component of the PCS scale were significant predictors for presence of bleeding gums (Table 3). Only VAS scores (*p*=0.036) were significant predictors of presence of periodontal pocket in the study population ( Table 4).

**Table 3:**
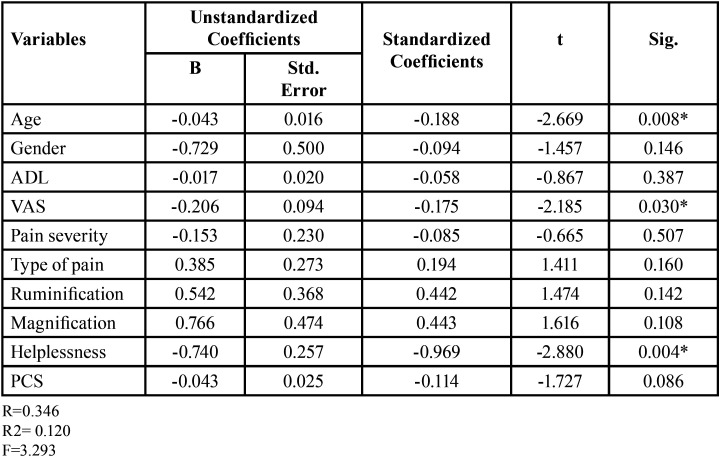
Linear Regression analysis for Presence of Gingival Bleeding.

**Table 4:**
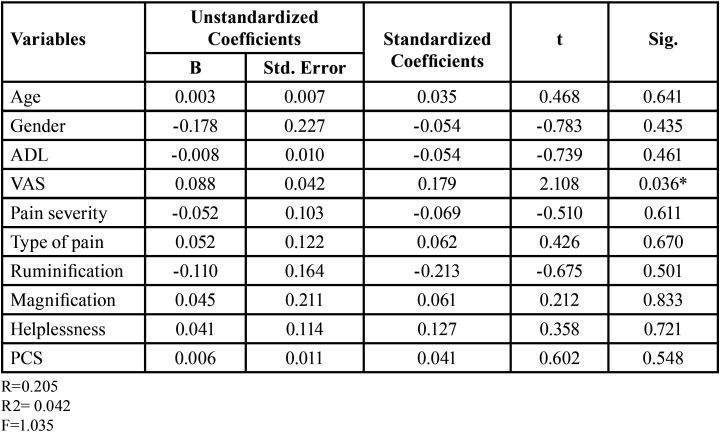
Linear Regression analysis for Presence of Periodontal Pockets.

## Discussion

The purpose of the present study was to examine pain catastrophizing among individuals with physical disabilities during oral disease. Although, a consistent relation has been observed between catastrophizing and pain under a variety of aversive conditions, it remains unclear whether catastrophizing contributes to pain experience during dental problems.

This study has provided a valuable insight into the oral health of people with physical disability. Assessment of ADL is important when measuring the disease severity, evaluating interventions and determining care needs in individuals with physical disability. However, there is currently no general consensus regarding instruments for ADL evaluation in patients with physical disabilities. Although, some instruments have been specifically designed to assess ADL in such individuals, they being time consuming and having incomplete validation restrict their use in clinical practice or epidemiological studies (29,30). The ADL index is a frequently used measure of independence in the activities of daily living (ADL).

The Barthel index (BI) is a widely used measure of basic ADL function (self- maintenance skills such as dressing, bathing, and grooming) because of its simplicity, communicability and ease of scoring. Similar to the other ADL instruments, the items of BI possess a hierarchy of difficulty and yield ordinal intervals between the scores and are supposed to have good validity when compared to the others (25).

The mean ADL of the study population was 80.5±12.5 i.e., majority of them belonged to moderate dependency levels which states that the individuals had relatively high degree of impairment requiring physical assistance from the caregivers in their daily activities.

The pain catastrophizing scale is a psychological instrument developed by Sullivan and Bishop (27) which objectifies catastrophizing associated with pain. PCS is a reliable and valid measure of catastrophizing. Catastrophizers tendency to focus on pain sensations may interfere with the effective use of pain sensations strategies. Thereby, in this study pain was assessed using Short form McGill pain (26) questionnaire which is considered to be a validated pain assessment tool particularly suiTable for situations where qualitative pain assessment is required, but time to collect is restricted.

Sensory pain and excruciating type of pain was the main pain reported by the population. Significant level of pain catastrophizing was seen only among 4.8% of the population, the overall mean PCS score of the population was 5.8±9.8. Jin Ho *et al*. (26) reported the mean PCS score of 17.3± 12.26 for 155 temporo mandibular joint disorder patients in Korea. Taking these together, patients with dental pain appear to have a very lower level of catastrophizing than that of the other pain conditions although catastrophizing level cannot be simply compared across the different samples because of possibility that there are influencing factors such as age, gender, etc. The mean DMFT score of the population in the present study which was 2.8±4.09, the DMFT score observed in our study was found to be lower than a previous report by Pradhan *et al*. (16) among Australian adults (5.2±4.5). Also, Orsos *et al*. (9) showed that in Hungarian individuals with physical disability had high caries experience (18.90±7.85), similarly Lee at al. (18) found that individuals with disability had adverse oral health than the non disabled person.

A significant positive correlation was observed between DMFT values and PCS suggesting that individuals with decayed teeth would have experienced and attributed to avoidance behaviour towards dental care. On the other hand, bleeding gums was negatively correlated with sensory pain and PCS, indicating that those individuals with higher dependency levels might be relying on a care taker for their oral hygiene practices.

Since, this group of population is already compromised owing to their disability; a multidimensional approach (both physical and psychological) to addressing pain catastrophizing is indispensable. Considering the severity of functional impairments in these individuals, seeking health care services is mainly dependent on the caregiver’s decision. Moresover, pain catastrophization is influenced by various personal and social factors such as past experiences with pain, social support, cultural beliefs about pain and health care access, efforts should be undertaken to provide information to care givers at family homes regarding dental services available through support organizations so as to enable them for routine dental care.

Use of all standardized instruments (25-28), oral examination conducted by pretrained and precalibrated dentists and calculation of sample size contribute to the strengths of the study. However, single institute study and cross sectional study design are the limitations of this study.

## Conclusions

A significant positive correlation was observed between higher DMFT values and higher PCS levels (r=0.45, *p*=0.000), whereas bleeding of gums was negatively correlated with sensory pain (r=-0.13, *p*=0.049) and PCS (r= -0.194, *p*=0.004). Periodontal pocket of less than 6mm was negatively correlated with ADL scores (r= -0.135, *p*=0.046).

Apart from this, pain catastrophization also leads to frequent visits to health care providers seeking relief or reassurance, thereby burdening the health care resources. Strategies like education about pain and disability, social support programs can help to instill a positive mindset, cultivating resilience which play a crucial role in mitigating the impact of pain catastrophizing especially among disabled individuals. Also, potential interventions, targeting reduction in catastrophizing might benefit in diminishing sensitivity to acute and chronic pain as well as buffering the deleterious effects of pain in quality of life for community at large.

Encouraging prospective research and identifying the clinical challenges can help to develop cost- and time-effective means to identify individuals who catastrophize and implement interventions to reduce their level of distress which might result in enhanced patient compliance.
